# The Interaction of Microalgae Dietary Inclusion and Forage-to-Concentrate Ratio on the Lipid Metabolism-Related Gene Expression in Subcutaneous Adipose Tissue of Dairy Goats

**DOI:** 10.3390/ani14223291

**Published:** 2024-11-15

**Authors:** Panagiota Kyriakaki, Alexandros Mavrommatis, Eleni Tsiplakou

**Affiliations:** Laboratory of Nutritional Physiology and Feeding, Department of Animal Science, Agricultural University of Athens, Iera Odos 75, GR-11855 Athens, Greece; kyriakaki@aua.gr (P.K.); mavrommatis@aua.gr (A.M.)

**Keywords:** dairy goats, polyunsaturated fatty acids, microalgae, *Schizochytrium* spp., forage-to-concentrate ratio, adipose tissue, tail fat, lipid metabolism, gene expression

## Abstract

Long-chain polyunsaturated fatty acids (PUFAs) such as docosahexaenoic acid (DHA) have been shown to be effective in enhancing the quality of ruminant products, including meat and milk. However, under these dietary conditions, the de novo lipogenesis could be influenced, too. On the other hand, even if the forage-to-concentrate ratio (F:C) is also a key factor affecting lipid metabolism in small ruminants, there is scarce information about its interaction with dietary PUFA. This study investigates the potential of the F:C ratio as a lever to manipulate lipid metabolism in dairy goats under high dietary PUFA supplementation. For this purpose, twenty-two crossbred dairy goats [Alpine × Local (Greek) breeds] (BW = 50.6 ± 6.1 kg) at early lactation (70 ± 10 days in milk) during the age of 3–4 years old, were separated into two homogeneous subgroups (n = 11). In the first phase, each goat was fed 20 g *Schizochytrium* spp./day followed by either a high-forage (20 HF) or a high-grain (20 HG) diet, while in the second phase, each goat was fed 40 g *Schizochytrium* spp./day followed once again either a high-forage (40 HF) or a high-grain (40 HG) diet. The F:C ratio of a high-forage and high-grain diet was 60:40 and 40:60, respectively. Tail fat tissue samples were collected by biopsy on the 42nd day of each experimental phase (last day). Significant decreases (*p* < 0.05) in the gene expression of *ACACA*, *CBR2*, *COX4I1*, *ELOVL5*, *ELOVL7*, *LEP,* and *SCD* were presented in goats fed 40 g compared to those fed 20 g *Schizochytrium* spp., while the gene expression of *ACACA*, *AGPAT2*, *AGPAT3*, *ELOVL5*, *ELOVL6*, *EPHX2*, *FASN*, and *SCD* was decreased in high grain compared to high-forage diets. This study also indicated that with the aim to enrich goat products with PUFA by increasing their levels in the diet, lipid metabolism is negatively affected. However, a diet with higher forage inclusion can partially attenuate this condition.

## Simple Summary

Polyunsaturated fatty acids (PUFAs), especially those obtained from microalgae, are an efficient and sustainable strategy to enrich ruminant products with biofunctional molecules. However, the high dietary inclusion needed to substantially affect product quality can also bring negative effects on lipid metabolism. The dietary forage-to-concentrate ratio (F:C) is also a lever to drive metabolic functions, including lipid metabolism, with the advantage of its easy manipulation. To address this hypothesis, we conducted an experiment assessing the interaction of two inclusion levels of *Schizochytrium* spp. (20 or 40 g/day), a microalga rich in PUFA, and two F:C ratios (60/40 or 40/60) on regulating gene networks involved in lipid metabolism in goats’ tail fat. Results showed a decreasing gene expression under the effect of high microalgae levels or the high-grain diet. However, when the diet was based on forages, the negative effect of high microalgae levels on lipogenic gene expression was more attenuated.

## 1. Introduction

Adipose tissue, along with the liver and mammary gland in lactating ruminants, are the principal sites of the de novo synthesis of fatty acids (FA) [1]. The significance of adipose tissue lies in the energy provision, which accounts for up to 10% of milk fat synthesis, apart from periods of negative energy balance, which is higher [2]. On the other hand, the subcutaneous adipose tissue plays an important role during the lactation period, as it is responsible for the mobilization of stored lipids to provide the energy needed for milk FA synthesis [3]. The de novo synthesis is driven by the synergistic effect of key lipogenic genes, with the most significant of them being Acetyl-CoA Carboxylase Alpha (*ACACA*) and Fatty Acid Synthase (*FASN*), while, in the case of adipose tissue and mammary gland, the Lipoprotein Lipase (*LPL*) also participates in the uptake of FA found in plasma [4]. Additionally, FA could be released from triglycerides (TAGs) or very-low-density lipoproteins (VLDL) and through the action of Stearoyl-CoA Desaturase (SCD) (Δ9-desaturase) could be desaturated in cis-9-unsaturated FA [4].

Gene expression is affected by many factors, including genetic, nutritional, and environmental ones. From a nutritional perspective, the consumption of docosahexaenoic (DHA) and eicosapentaenoic (EPA) acid in humans is considered to have a positive effect on white adipose tissue function and metabolism [5] by activating the transcription factor *PPARα*, increasing transcription of lipolytic genes, and decreasing transcription of lipogenic genes [6]. The mechanism by which the ω-3 FA suppresses the expression of lipogenic genes is not fully described yet. It seems that ω-3 FA could decrease the expression of sterol regulatory element-binding protein 1c (*SREBP-1c*) inhibiting phosphatidic acid phosphatase (PAP) and 1,2-diacylglycerol acyltransferase (DGAT) [7].

Considering animal species, the diet effect on gene expression in adipose tissue is mainly focused on mice and pigs, while recent data for ruminants are scarce. Dietary polyunsaturated FAs (PUFAs) are negatively correlated to *de novo* FA synthesis [8,9,10], while it has been reported that ω-3 rich diets modify serum and adipose lipid profiles [11]. This downregulation could be attributed to the inhibitory effect of ω-3 and ω-6 PUFA on the expression of receptor subfamily 1, group H, member 3 (*NR1H3*) [10]. A study of NR1H3^(−/−)^ in mice showed a reduction in the de novo FA synthesis due to the downregulation of *SREBF1* and its target genes [12]. A PUFA-rich diet inhibited the lipogenic activity in pigs, providing connection evidence between lipogenic gene expressions and FA accumulation in their adipose tissue [13]. A 68% reduction in the *SCD* mRNA abundance in the tail-head adipose tissue of soybean (rich in linoleic acid)-fed goats was found, while the mRNA abundance of *LPL*, *ACACA*, and *FASN* was not significantly altered in any of the other studied lipogenic tissues [14]. Moreover, Joseph et al. [15] found that supplementing steers’ diet with corn oil (rich in linoleic acid) at 0.31 kg/d upregulated the expression of *FASN*, *SCD*, and *LPL* genes in tail-head adipose tissue, while that of 0.62 kg corn oil/d downregulated the former genes, highlighting a severe dosage effect. Lastly, the dietary inclusion of olive oil (rich in oleic) in dairy cows did not affect the expression of genes involved in de novo fatty acid synthesis in tail-head adipose tissue [16]. Several microalgal species have been recorded as sources of PUFAs [17]. *Schizochytrium* is a single-cell heterotrophic marine microbe independent of sunlight for growth and with an outstanding ability to accumulate FA. *Schizochytrium* is extensively characterized for DHA production due to unique enzymes catalyzing elongation and desaturation, reaching a lipid content ranging from 42% to 67% in a 96 h fermentation [18]. Dietary supplementation with *Schizochytrium* spp., rich in ω6-Docosapentaenoic acid (ω6-DPA) and DHA, has been introduced aiming to enrich ovine [19] and caprine [20,21] and bovine [22] milk with DHA, ω6-DPA, and conjugated linoleic acid (CLA). Additionally, dietary supplementation with *Schizochytrium* spp. increased DHA accumulation in both adipose and muscle tissues of growing lambs [23].

Further to the impact of dietary PUFA inclusion in lipid metabolism, the F:C ratio of the diet affects energy efficiency [24] and fat deposition [25] as well. However, their interaction in lipogenic gene networks has not been studied to the best of our knowledge. Considering the above-mentioned issues, it is plausible to assume that by increasing the levels of PUFA-rich sources in ruminants’ diets aiming to fortify products with bioactive molecules, lipid metabolism could be negatively affected through the downregulation of key *de novo* lipogenic genes. The simultaneous manipulation of the F:C ratio may be an easy and feasible strategy to redirect lipid metabolism. Thus, the present study aimed to investigate the effect of two inclusion levels of *Schizochytrium* spp. (20 or 40 g/day) and two F:C ratios (60/40 or 40/60) on regulating gene networks involved in lipid metabolism in goats’ tail fat adipose tissue.

## 2. Materials and Methods

### 2.1. Experimental Design

This research follows the experimental design of previously published studies [20,21,26]. This study was conducted according to the guidelines of the European Union Directive on the protection of animals used for scientific purposes (EU 63/2010; Council of the European Union 2010), and the report of the Agricultural University of Athens Ethical Committee in Research. Briefly, twenty-two crossbred dairy goats [Alpine × Local (Greek) breeds] at early lactation (70 ± 10 days in milk) at the age of 3–4 years old were divided into two homogenous groups (n = 11) according to their body weight (BW = 50.6 ± 6.1 kg) and fat-corrected milk yield (FCM4%). The experimental trial was separated into two phases, with two dietary groups per phase. More specifically, in the first phase, each goat was fed 20 g *Schizochytrium* spp./day, followed by either a high-forage (20 HF) or a high-grain (20 HG) diet, while in the second phase, each goat was fed 40 g of *Schizochytrium* spp./day, followed once again by either a high-forage (40 HF) or a high-grain (40 HG) diet. The F:C ratio of high-forage (HF) and high-grain (HG) diets was 60:40 and 40:60, respectively, whereas forages (alfalfa hay and wheat straw) were provided separately from the concentrate in the feeders in two equal portions after milking. The former F:C ratios were selected, aiming to be applicable in commercial small ruminant farms. More specifically, HF goats were offered 1 kg of concentrate, 0.3 kg of wheat straw, and 1.2 kg of alfalfa hay, while HG goats were offered 1.3 kg of concentrate, 0.18 kg of wheat straw, and 0.7 kg of alfalfa hay, per goat, daily. Each phase lasted 8 weeks, of which the first 2 were considered as an adaptation period. Animals were fed on a group basis, with their average energy and nutritional requirements and diets formulated to be balanced based on the National Research Council [27]. Each dietary group was housed in a three-sided enclosed unit, with the southern side semi-open and the ceiling thermally insulated. The experiment was conducted from March to June 2015, with temperatures ranging from 12 to 26.4 °C, relative humidity between 45% and 66%, and cumulative rainfall totaling 90.9 mm. *Schizochytrium* spp. was supplied as a commercial product labeled DHA gold (DSM Nutritional Products, Marousi, Greece), which was added to the concentrate to provide 20 and 40 g/goat/day in both high-forage and high-grain diets, as shown in Table 1. Diet consumption was being recorded on a daily basis and all animals had free access to fresh water.

### 2.2. Milk Sampling

Goats were milked twice per day (08:00 and 17:00). Individual milk samples were collected on the 7th, 14th, 21st, 28th, 35th, and 42nd days of each experimental phase in order to determine their chemical composition, while milk samples on the 21st and 42nd days were used for studying its fatty acid profile (Table S1). Before both analyses, samples from the morning and evening milking were pooled at a percent volume of 5% for the highest reliability. All these results are already published in the study of Mavrommatis et al. [21]. Lactation data were used in this study only for correlation with gene expressions.

### 2.3. Tail Tissue Sampling

Tail adipose tissue was taken with a biopsy from the tail-head region on the last day of each dietary phase after morning milking. Before biopsies, the hair on the tail head was clipped closely and thoroughly scrubbed with surgical soap. A xylocaine 2% (2 mL lidocaine hydrochloride, AstraZeneca, Athens, Greece) local anesthesia was also provided to goats. A 2–3 cm incision with a scalpel was made and the skin was pulled back using sterile forceps, exposing the adipose tissue. About 150 mg of adipose tissue was collected under sterile conditions, immediately frozen in liquid nitrogen, and stored at −80 °C until RNA extraction. After the biopsy, the sampling site was cleaned with a disinfecting chlorhexidine powder (Terramycin, w/Polymyxin, Pfizer, Athens, Greece) and with a spray (Oxyvet, Provet, Athens, Greece) to prevent the after-biopsy infections. Animals were fully recovered from tail tissue sampling and kept at the experimental unit.

### 2.4. RNA Isolation, DNAse Treatment and Cleaning, and cDNA Synthesis

Total RNA was extracted from 150 mg of tail tissue using Trizol reagent (Invitrogen, Paisley, UK), while afterward, chloroform and isopropanol were added according to the manufacturer’s protocol. The RNA pellet was diluted in RNAse-free water (HyPure Molecular Biology Grade Water, Cytiva, South Logan, UT, USA). In order to prevent DNA contamination, RNA was initially treated with 2 units of DNAse (Invitrogen, Thermo Fisher Scientific, Waltham, MA, USA), remaining at 35 °C for 15 min. For the next step, the NucleoSpin^®^ RNA Plus kit (Macherey-Nagel GmbH & Co., KG, Düren, Germany) was used. Primarily, the filtrate was treated with the Lysis Buffer, Binding Solution, and absolute ethanol (100%). Subsequently, all the quantity was transferred to the RNA Plus Column, followed by washes with WB1 and WB2 according to the manufacturer’s protocol. Finally, 40 μL of RNAse-free water was added to the column, and after a centrifuge at 11,000× *g* for 1 min, RNA was resuspended. A 2% agarose gel electrophoresis (Bio-Rad Laboratories Inc., Hercules, CA, USA) was performed to assess RNAs’ integrity at 80 V for 15 min. Discrete bands representing 28 s and 18 s ribosomal RNAs, respectively, were presented, demonstrating minor or no RNA hydrolysis. RNA was then quantified with a spectrophotometer (NanoDrop ND-1000, Thermo Fisher Scientific, Waltham, MA, USA) and qualified by the ratios A260/A280 and A260/A230. After assessing the quality of the extracted RNA, 6 out of 44 samples were excluded from further analysis. As a result, the 20 HF, 20 HG, 40 HF, and 40 HG groups consisted of 8, 10, 10, and 10 samples, respectively. Approximately 500 ng of pure RNA of each sample was reverse transcribed with the PrimeScript First Strand cDNA Synthesis Kit (Takara, Shiga, Japan) according to the manufacturer’s instructions using a mix of random hexamers and oligo-dT primers.

### 2.5. Determination of Relative Transcript Levels

Target genes were selected considering the gene networks involved in lipid metabolism in response to PUFA dietary supplementation [28]. Primers for Acetyl-CoA Carboxylase Alpha (*ACACA*), Acyl-CoA Oxidase 1 (*ACOX1*), 1-Acylglycerol-3-Phosphate O-Acyltransferase 1, 2, 3, 4, and 5 (*AGPAT1*, *AGPAT2*, *AGPAT3*, *AGPAT4*, and *AGPAT5*), AKT Serine/Threonine Kinase 2 (*AKT2*), Carbonyl reductase [NADPH] 2 (*CBR2*), Cytochrome C Oxidase Subunit 4I1 (*COX4I1*), elongation of Very-long-chain Fatty Acid-like Fatty Acid Elongase 1, 2, 3, 4, 5, 6, and 7 (*ELOVL1*, *ELOVL2*, *ELOVL3*, *ELOVL4*, *ELOVL5*, *ELOVL6*, and *ELOVL7*), Epoxide Hydrolase 2 (*EPHX2*), Fas Associated via Death Domain (*FADD*), Fatty Acid Synthase (*FASN*), Leptin (*LEP*), Lipoprotein Lipase (*LPL*), Prostaglandin-Endoperoxide Synthase 1 and 2 (*PTGS1* and *PTGS2*), and Stearoyl-CoA Desaturase (*SCD*) were designed by Geneious software 9.1.8. version (Biomatters Ltd., Auckland, New Zealand) according to the respective *Capra hircus* gene coding sequences (CDS in GenBank) (Table 2). The relative mRNA expression levels for the target genes were quantified with a StepOnePlus™ Real-time PCR System (Applied Biosystems, Foster City, CA, USA) using SYBR Select Master Mix (Applied Biosystems, Austin, TX, USA), gene-specific primers at a final concentration of 0.2 μM each (forward and reverse) and 1 μL of each cDNA as template. PCR cycling started at 95 °C for 15 min followed by 40 cycles at 95 °C for 15 s and 60 °C for 1 min. Primers’ specificity and the formation of primer dimers were monitored by melt curve analysis and by loading the amplification products to agarose gel electrophoresis to verify the production of a single amplicon 70–109 bp per reaction.

The geometrical mean of the expression levels of Glyceraldehyde 3-Phosphate Dehydrogenase (*GAPDH*), Actin Beta (*ACTB*), and Hypoxanthine Phosphoribosyl Transferase (*HPRT*) was used as housekeeping genes to normalize the cDNA template concentrations. Also, housekeeping genes were evaluated using BestKeeper [29], which indicated significant linear regression (*p* < 0.001) between the above reference genes. The relative expression levels of studied genes were calculated as (1 + E)^−ΔCt^, where ΔCt is the difference between the geometric mean of the three housekeeping genes’ Cts and the Ct of the target gene. Primer efficiencies were calculated according to target genes’ standard curves.

### 2.6. Statistical Analysis

SPSS 26.0 statistical package (IBM, Chicago, IL, USA) was used for data analysis. The effect of both factors, namely the microalgae level inclusion (20 or 40 g *Schizochytrium* spp.) and the F:C ratio (60:40 or 40:60), as well as their interaction effect on the transcript levels of the genes, was evaluated using multivariate analysis of variance (MANOVA). The significance tests were based on the linearly independent pairwise comparisons among the estimated marginal means. The simultaneous effect of the four diets (20 HF, 20 HG, 40 HF, and 40 HG) on the transcript levels of the genes was also evaluated using one-way ANOVA, followed by a post hoc analysis using the LSD multiple range test (Table S2). Different superscript letters signify statistical differences emerging by the post hoc analysis. For all tests, statistical significance was determined at an alpha level of *p* < 0.05, while 0.05 ≤ *p* < 0.10 was considered a tendency.

Furthermore, gene expression levels were correlated amongst them and further correlated with milk yield and chemical composition as well as the fatty acid profile, using the Spearman correlation. All these correlations are presented as heatmap graphs, while the R values are presented in the Supplementary Materials (Tables S3–S5). Discriminant plots were also generated (the independent-together method) regarding the relative transcript levels of lipogenic genes to investigate their classification amongst the four dietary treatments (20 HF, 20 HG, 40 HF, and 40 HG). For the assessment of discriminant functions, Wilk’s lambda (λ) criterion was used. Pool data of the gene expression of the thirty-eight samples were entered to discriminate the four groups. Lastly, a Euclidean distance analysis was applied to the means of twenty-five targeted gene expressions of the four dietary treatments (20 HF, 20 HG, 40 HF, and 40 HG).

## 3. Results

### 3.1. Feed Intake and Animal Performances

The intake of wheat straw was decreased by 34% and 50% in the 20 HF and 40 HF-fed goats, respectively (Table 3). The intake of the concentrate was also decreased in both 40 HF- and 40 HG-fed goats by 16%. These changes also decreased the microalgae intake since they have been supplemented into the concentrate (40 HF: 33.7 g and 40 HG: 33.2 g vs. the planned 40 g; Table 3). However, the planned F:C ratios and Neutral Detergent Fiber (NDF) to starch proportion were not substantially altered (Table 3). Overall, a high-grain diet combined with high microalgae levels resulted in lower Dry Matter Intake (DMI), and thus lower Protein Digestible in the Small Intestine (PDI) and energy consumption (Table 3). The mean body weight of goats did not differ among the dietary groups in both experimental phases. Milk yield and chemical composition are available in Table S1.

### 3.2. Gene Expressions

The effect of the F:C ratio and microalgae (*Schizochytrium* spp.) inclusion level, as well as their interaction effect on the relative expression of genes implicated in lipid metabolism of adipose tissue in goats’ tail fat, are presented in Table 4. The relative transcript levels of most genes were downregulated in tail adipose tissue of high-grain-fed (F:C = 40:60) compared to high-forage-fed (F:C = 60:40) goats, as well as in the groups supplemented with 40 g compared to 20 g *Schizochytrium* spp. (Table 4). More specifically, in regards to the F:C ratio, significantly lower relative transcript levels were observed for *ACACA* (*p* = 0.041), *AGPAT2* (*p* = 0.016), *AGPAT3* (*p* = 0.003), *ELOVL5* (*p* = 0.019), *ELOVL6* (*p* = 0.002), *EPHX2* (*p* = 0.009), *FASN* (*p* = 0.003), and *SCD* (*p* = 0.001) genes in tail adipose tissue of high-grain-fed (F:C = 40:60) compared to high-forage-fed (F:C = 60:40) goats (Table 4). Considering the microalgae inclusion level, the relative expression of *ACACA* (*p* = 0.002), *CBR2* (*p* = 0.021), *COX4I1* (*p* = 0.001), *ELOVL5* (*p* = 0.005), *ELOVL7* (*p* = 0.039), *LEP* (*p* = 0.038), and *SCD* (*p* = 0.005) was significantly decreased in the tail fat of goats fed with 40 g compared to 20 g of *Schizochytrium* spp. (Table 4). Lastly, results showed that there was a significant interaction between the two factors (F:C ratio and microalgae inclusion level) for *ACACA* (*p* = 0.005) and *AGPAT5* (*p* = 0.037) gene relative expression in the tail fat (Table 4).

Furthermore, it was also important to analyze the effect of both microalgae and F:C ration across the four dietary treatments (Table S2). Results showed a strong downregulation in the relative transcript levels of *AGPAT2* (*p* = 0.018), *AGPAT4* (*p* = 0.024), *ELOVL3* (*p* = 0.005), *ELOVL5* (*p* = 0.009), *EPHX2* (*p* = 0.006), *FASN* (*p* = 0.017), and *LEP* (*p* = 0.025) in the 40 HG group compared to groups fed with 20 g *Schizochytrium* spp., while *AGPAT3* (*p* = 0.043), *ELOVL6* (*p* = 0.005), and *SCD* (*p* = 0.003) were downregulated in both 40 HF and 40 HG groups (Table S2). On the other hand, the relative transcript level of *CBR2* (*p* = 0.038) was downregulated in the 20 HG group (Table S2). Lastly, a decreasing and an increasing trend were observed for the relative transcript levels of *ACACA* (*p* = 0.057) and *ACOX1* (*p* = 0.098), respectively, in the 40 HG group compared to groups fed with 20 g *Schizochytrium* spp. (Table S2).

### 3.3. Correlations with Animal Performances

**Figure 1:**
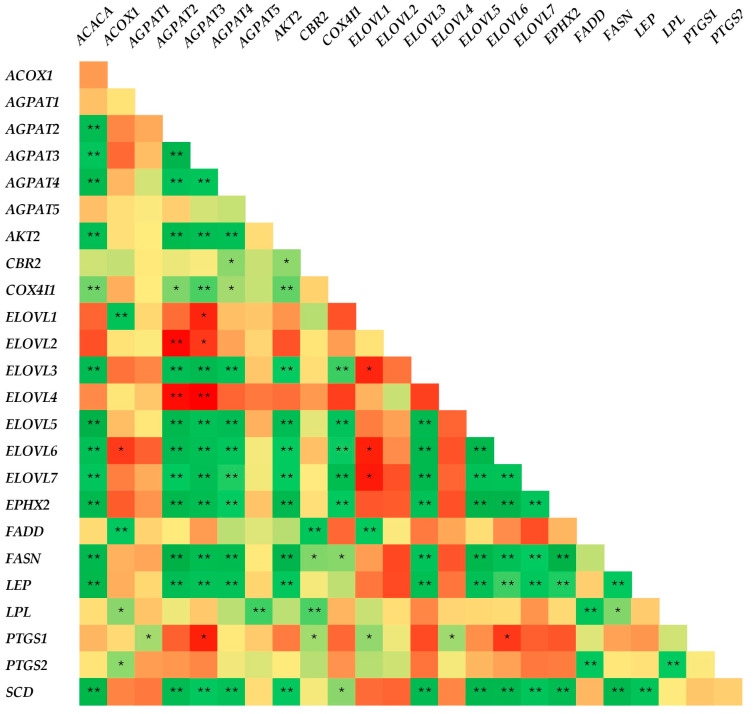
Spearman’s correlation heatmap between the mRNA expression of genes involved in lipid metabolism in goats’ tail fat. R values for each correlation are available in Supplementary Materials (Table S3). Darker colors indicate stronger correlations, while lighter colors indicate weaker correlations. Positive correlations are represented by green color, while negative correlations are represented by red color. * *p* < 0.05, ** *p* < 0.01.

Figure 1 presents the correlations between the mRNA expression of genes involved in lipid metabolism in goats’ tail fat. According to the Spearman correlation heatmap (Figure 1), most of the significant (*p* < 0.05) correlations found were positive, apart from *AGPAT3,* which was negatively correlated with *ELOVL1*, *ELOVL2,* and *PTGS1* (*p* < 0.05), as well as *ELOVL4* (*p* < 0.01), *AGPAT2,* which was negatively correlated with *ELOVL2* and *ELOVL4* (*p* < 0.01), *ELOVL6,* which was negatively correlated with *ACOX1*, *ELOVL1,* and *PTGS1* (*p* < 0.05), and *ELOVL1,* which was also negatively correlated with *ELOVL3* and *ELOVL7* (*p* < 0.05).

**Figure 2:**
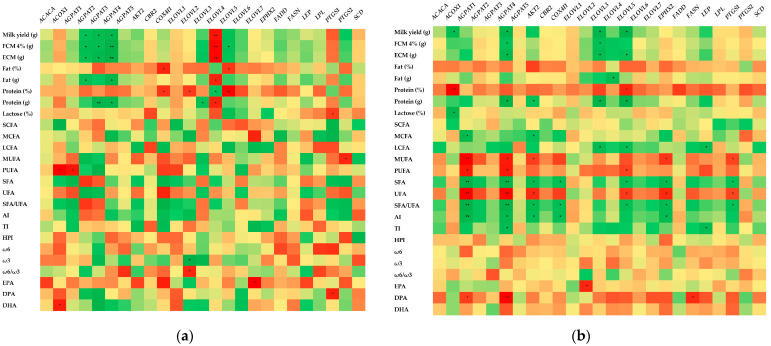
Spearman’s correlation heatmap of the mRNA expression of genes involved in lipid metabolism in goats’ tail fat and milk performance (milk yield, fat, protein, and lactose) or its fatty acid profile at (**a**) first experimental phase; (**b**) second experimental phase. R values for each correlation are available in Supplementary Materials (Tables S4 and S5). Darker colors indicate stronger correlations, while lighter colors indicate weaker correlations. Positive correlations are represented by green color, while negative correlations are represented by red color. * *p* < 0.05, ** *p* < 0.01.

Figure 2 presents correlations between the mRNA expression levels and milk performances (milk yield, fat, protein, and lactose) and milk fatty acid profile in each experimental phase. In the first experimental phase, milk yield as well as fat and protein content were positively correlated (*p* < 0.05) with *AGPAT2*, *AGPAT3,* and *AGPAT4* gene expression, while the expression of the *ELOVL4* gene seemed to be negatively correlated (*p* < 0.05) with the milk performance factors (Figure 2a). Furthermore, PUFA content was negatively correlated (*p* < 0.05) with *ACOX1* and *AGPAT1* gene expression, while EPA, DPA, and DHA content were negatively correlated with *ELOVL7*, *PTGS1,* and *ACOX1*, respectively (Figure 2a). On the other hand, in the second experimental phase, milk performance parameters did not significantly affect the expression of most lipogenic genes. Milk yield was positively correlated (*p* < 0.05) with *AGPAT4*, *ELOVL3*, and *ELOVL5* gene expression, while fat and protein content were positively correlated (*p* < 0.05) with *ELOVL4* and *AGPAT4*, *AKT2*, *ELOVL3*, and *ELOVL5*, respectively (Figure 2b). Additionally, *AGPAT1, AGPAT4,* and *ELOVL5* were negatively correlated with PUFA content (*p* < 0.05), while the atherogenicity index (AI) was positively correlated with *AGPAT1* (*p* < 0.01), *AGPAT4*, *AKT2*, *COX4I1*, and *EPHX2* (*p* < 0.05) (Figure 2b). On the other hand, the thrombogenicity index (TI) was positively correlated with *AGPAT4* and *LEP* (*p* < 0.05) (Figure 2b). Lastly, EPA was negatively correlated (*p* < 0.05) with *ELOVL2,* whereas DPA was negatively correlated (*p* < 0.05) with *AGPAT1, FASN* (*p* < 0.05), and *AGPAT4* (*p* < 0.01) gene expression (Figure 2b).

**Figure 3:**
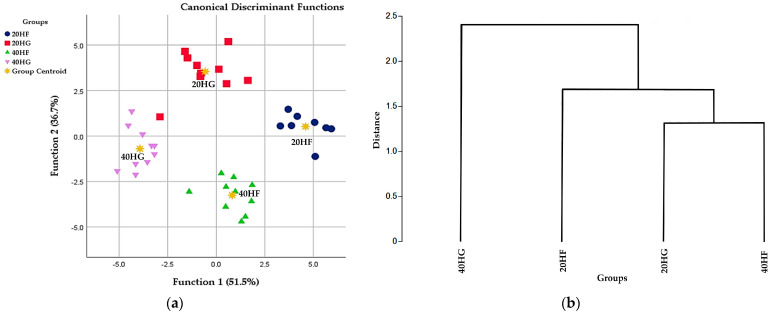
(**a**) Discrimination analysis and (**b**) Euclidean distance diagram of the four dietary groups; 20 HF:20 g *Schizochytrium* spp./day and F:C = 60:40; 20 HG:20 g *Schizochytrium* spp./day and F:C = 40:60; 40 HF:40 g *Schizochytrium* spp./day and F:C = 60:40; 40 HG:40 g *Schizochytrium* spp./day and F:C = 40:60, based on the pooled data of relative transcript levels of key genes implicated in lipid metabolism of adipose tissue in goats’ tail fat.

Discriminant analysis was also applied to the pooled data of relative transcript levels to establish those variables capable of distinguishing and classifying samples among the four (20 HF, 20 HG, 40 HF, 40 HG) dietary groups (Figure 3a). Wilks’ lambda (λ) was reported at 0.004 for Function 1 (*p* < 0.001) and 0.038 for Function 2 (*p* = 0.011), indicating clear discrimination, while the relative transcript levels of *ELOVL3*, *EPHX2*, *ELOVL5*, *SCD*, *LEP*, *FASN*, *ELOVL6*, *AGPAT2*, *ACACA*, *COX4I1*, and *ELOVL7* were the variables that contributed the most (Figure 3a). Euclidean distance analysis revealed a higher similarity in lipid metabolism gene expressions between goats fed with 20 HG and 40 HF diets, whereas those fed with the 40 HG diet exhibited the lowest (Figure 3b). 

## 4. Discussion

The high microalgae level (40 HF and 40 HG) in goats’ diet led to decreased feed concentrate intake and, consequently, reduced microalgae consumption compared to the planned one. The lower concentrate intake may have been impaired by the fish-like flavor of the *Schizochytrium* spp. or the elevated fat content in the 40 HF and 40 HG due to cholecystokinin’s effect [30]. Wheat straw was also decreased in high-forage diets by up to 50% due to its low palatability and animal resistance to long particles [31]. However, the formulation of the experimental diets using straw was the least intrusive way to achieve isonitrogenous, isocaloric, and different F:C ratios at the same time. It should be mentioned here that, from here on, all assumptions regarding the dietary group 40 HG should also consider lower energy and protein intake as a co-factor.

Even though there is an increasing interest in fortifying ruminants’ products with PUFAs for the benefit of human health, scarce information exists about the influence of microalgae in the expression of genes participating in the lipid metabolism of adipose tissue. Also, it is noteworthy that no data exist for goats, while research works on tail fat are limited. The increased microalgae and concentrates levels in goats’ diet affected the majority of genes involved in fatty acid elongation (e.g., *ELOVL* genes) and the de novo synthesis (e.g., *FASN*, *ACACA*), whereas it seems that changes in the F:C ratio also influenced the expression of genes involved in triglycerides synthesis (e.g., *AGPAT* genes). The fact that the forage-to-concentrate ratio also affects lipid metabolism gene expressions highlights the opportunity to manipulate the dietary NDF/starch ratio, aiming to attenuate any reverse effect of PUFA overload in small ruminants’ diets. This hypothesis was supported by the distance analysis, which exhibited that the goats consuming 33.7 g *Schizochytrium* spp. in a diet with F:C = 60:40 had a comparable gene expression profile with those supplemented with 20 g *Schizochytrium* spp. and F:C = 40:60 diet. Considering at the same time the effect of high PUFA content in the 40 HG-fed goats, it is plausible to conclude that the high-forage diet attenuated the adverse impact of PUFA in gene expressions.

In the past, studies that focused on rodents have demonstrated that PUFA supplementation mostly suppresses the mRNA levels of lipogenic genes in adipose tissue and the liver [32,33], while studies on gene transcriptions showed that PUFAs upregulate PPARs, but downregulate LXRs and SREBPs [34]. However, PUFA supplements are not the only factor that possibly influences the lipid metabolism in ruminants, as there is evidence that differences between the basal diet could also play a pivotal role [15,35].

Supplementing cows’ diet with fish oil or microalgae (200 g/d) in a pasture or confinement system had no effect on the expression of *LPL*, *ACACA*, *FASN*, *SCD*, *FADS1*, and *FADS2* genes in subcutaneous adipose tissue, while the expression of *FASN*, *SCD*, *FADS1*, and *FADS2* genes was downregulated in their liver [36]. On the other hand, the inclusion of fish oil (1% DM) in cows’ diet upregulated significantly the mRNA abundance of *LPL, SCD*, *ADFP*, and *LPIN1* in subcutaneous adipose tissue, suggesting that these changes in gene expression are indicative of a greater lipid deposition [37]. Another study showed that the *SCD* expression was decreased in *Longissimus dorsi* of cows fed with fish (1 or 2%; 9 or 18 g/kg as fed) and soybean oil (6%; 60 g/kg as fed) simultaneously [38]. The dietary inclusion of *Schizochytrium* spp. (3.89% DM), along with extruded linseed (5% DM), downregulated the *ACACA*, *LPL*, and *SCD,* while it did not affect *FADS1*, *FADS2*, and *ELOVL5* in the subcutaneous adipose tissue of lambs [39]. Additionally, the transcription factors, *PPARγ* and *CEBPA,* were increased, while *SREBF1* remained unaffected in the treated animals [39]. In contrast, the expression of *ACACA*, *LPL*, *SCD*, *FADS1,* and *FADS2* genes was downregulated, while the *PPARγ*, *CEBPA,* and *SREBF1* remained unaffected in the intramuscular adipose tissue of treated lambs [39]. Moreover, the expression of *FADS2*, *ELOVL2*, *SCD*, *CPT1α,* and *SREBF-1* was upregulated, while that of *PPARα* and *PPARγ* was decreased in longissimus muscle of lambs fed with an algae powder from *Schizochytrium* spp. (3% DM) [40]. Additionally, the gene expression of *SCD* and *ACACA* in subcutaneous fat and *SCD* in the liver, as well as *CPT1* in both longissimus muscle and subcutaneous fat, was unaffected, while the expression of *FADS1* in the liver was increased with the algae (1.92% DM) inclusion [41]. Moreover, supplementing lambs’ diet with calcium salts of EPA + DHA (0.39% DM) downregulated the mRNA expression of *SCD* and *FASN* in the liver during the finishing period, suggesting a decreased lipogenesis and increased lipolysis in the liver of lambs [42]. On the other hand, the expression of *LEP* and *FASN* was upregulated in ewes fed EPA and DHA (7.82 g/ewe/day) compared to the calcium-salts treatment (source of palmitic and oleic acids) during late gestation [43]. According to Chilliard et al. [44], leptin is firmly related to body fat in ruminants as higher concentrations are observed in fat compared to lean animals. Hence, the upregulation in the expression of the *LEP* gene, along with the upregulation in the *FASN* gene, indicates greater fatty acid synthesis in tail fat tissue. Contrariwise, both genes were downregulated under the increasing level of microalgae dietary inclusion [45].

Alvarenga et al. [41] investigated the interaction between the algae inclusion (1.92% DM) and dam diet (silage or oat/cottonseed grain) during the conception period on the expression of lipogenic genes in lambs. They found that the expression of *ACACA*, *SCD*, *FADS1*, and *FADS2* was upregulated in the longissimus muscle of algae-silage-fed lambs, while the *FADS1* and *FADS2* gene expression was also upregulated in their subcutaneous adipose tissue [41]. An upregulation was also found for *FADS2* and *ACACA* gene expression, whereas the expression of the *CPT1* gene was decreased in their liver [41]. These results show that the inclusion of microalgae, along with the type of forage, differently affects the expression of lipogenic genes. In this context, the effect of the F:C ratio was also evaluated in the present study. According to the literature, a study comparing the effects of pasture, pasture with soybean hulls and corn oil, pasture with corn grain, or a high-concentrate diet concluded that the inclusion of both corn oil and corn grain affected the expression of genes involved in fatty acid synthesis in bovines [15]. Moreover, providing a high-concentrate diet to increase the dietary energy intake altered the transcription of genes encoding enzymes implicated in bovine fat metabolism, subsequently affecting the fatty acid content in the carcass tissue and the carcass quality [15]. A study by Daniel et al. [35] concluded that high-concentrate diets increased the expression of *SCD* genes compared to forage ones. Further, it has been shown that grass-feeding upregulates *SCD* gene expression associated with the higher converting ratio of MUFAs to SFAs [46]. Our previous work showed that sesame seeds rich in linoleic acid did not significantly affect the lipogenic gene expressions in the mammary gland of goats [47], while, in this study, both DPA and DHA dietary inclusion (even in lower inclusion levels) robustly downregulated liposynthesis in the tail adipose tissue of goats. Beyond the difference in tissue level, it seems that the type of fat strongly manipulates lipogenic pathways differently.

## 5. Conclusions

Increased levels of microalgae and concentrate in goats’ diet severely affect the expression of genes involved in fatty acid elongation (e.g., *ELOVL* genes) and de novo synthesis (e.g., *FASN*, *ACACA*) in tail fat tissue. This study reported that although high PUFA content in goats’ diet can substantially downregulate genes regulating lipid synthesis and metabolism in adipose tissue, replacing starch content by NDF through forage-to-concentrate ratio can attenuate this adverse effect. This is of significant importance to the dairy industry, as efforts to develop added-value dairy products fortified with PUFA and ω-3 fatty acids can often compromise milk composition. Manipulating the forage-to-concentrate ratio in PUFA-rich diets for dairy goats offers a practical, sustainable approach to regulate lipid metabolism and mitigate the adverse effects of PUFA overload on the animal.

## Figures and Tables

**Table 1:** Concentrate ingredients (g/kg) of the four diets (20 HF, 20 HG, 40 HF, and 40 HG).

	Diets			
	20 HF	20 HG	40 HF	40 HG
Concentrate (g/kg)				
Maize grain	387	331.6	357	326.3
Barley grain	200	200	200	200
Wheat middlings	210	210	210	210
Sunflower meal	80	80	80	80
Soybean meal	60	120	70	110
Calcium phosphate	15	15	15	15
Calcium carbonate	5	5	5	5
Salt	3	3	3	3
Mineral and vitamin	20	20	20	20
*Schizochytrium* spp.	20	15.4	40	30.7

20 HF: goats fed with 20 g *Schizochytrium* spp./day followed a high-forage (F:C = 60:40) diet; 20 HG: goats fed with 20 g *Schizochytrium* spp./day followed a high-grain (F:C = 40:60) diet; 40 HF: goats fed with 40 g *Schizochytrium* spp./day followed a high-forage (F:C = 60:40) diet; 40 HG: goats fed with 40 g *Schizochytrium* spp./day followed a high-grain (F:C = 40:60) diet.

**Table 2:** Sequences, amplicon size, and accession number of primers used in real-time qPCR.

	Forward Primer	Reverse Primer	Amplicon Size (bp)	Acc. No.
*GAPDH*	5′-AAAGGCCATCACCATCTTCCA-3′	5′-ACCACGTACTCAGCACCAGCAT-3′	75	XM_005680968.3
*ACTB*	5′-CCTTTGCCTTCCCAAAAGCC-3′	5′-AAGCAATCACCTCCCCTGTG-3′	87	XM_018039831.1
*HPRT*	5′-TATGGACAGGACCGAACGAC-3′	5′-AGAGGGCCACAATGTGATGG-3′	71	XM_018044253.1
*SCD*	5′-TGGCGTTCCAGAATGACGTT-3′	5′-GGGAATTGTGGGGATCAGCA-3′	90	NM_001285619.1
*ACACA*	5′-CCAACAATGGCATTGCAGCT-3′	5′-CGCACGCTCATTTCGAAACA-3′	80	XM_018064168.1
*FASN*	5′-AACCTGGAGGAGTTTTGGGC-3′	5′-GCCATATAGTCCCGCCTTCC-3′	87	NM_001285629.1
*LPL*	5′-TCTCAGGACTCCCGAAGACA-3′	5′-AGCCACAGATTCCGTCACTC-3′	70	NM_001285607.1
*LEP*	5′-CCTGGAAGCCTCCCTCTACT-3′	5′-GCCGCAACATGTCCTGTAGA-3′	77	XM_018046968.1
*ELOVL1*	5′-ATGTCCGGCTGGCTAAGTTC-3′	5′-GAACCATCCTCAGTGCCTCC-3′	82	XM_005678573.3
*ELOVL2*	5′-CCGGCCATGGAGCATCTAAA-3′	5′-GCATGAACCAGCCTCTGACT-3′	100	XM_018039308.1
*ELOVL3*	5′-TCTGAGGCTCTGGCTTGTTG-3′	5′-AAAGCTAGGGGACGGAGGAT-3′	92	XM_005698356.2
*ELOVL4*	5′-CGACACCGTGGAGTTCTACC-3′	5′-GACTGCATCAGAGGCCAGTT-3′	75	XM_018052902.1
*ELOVL5*	5′-GATCATCCGTGTGCTCTGGT-3′	5′-TGGTTGTTCTTGCGGAGGAT-3′	87	NM_001285628.1
*ELOVL6*	5′-AGCCTCTAGTGCTCTGGTCT-3′	5′-ATGCTTCAGGCCTTTGGTCA-3′	107	XM_005681307.3
*ELOVL7*	5′-TTGAGCATGGGCAGAGATCC-3′	5′-GCAACAAAGCCAAAGCCCAT-3′	86	XM_018065758.1
*CBR2*	5′-AAAGGGATTGGACGGGACAC-3′	5′-CACTCCTTGGAGAGGCTGAC-3′	104	XM_018065425.1
*EPHX2*	5′-AACTACCCCATGCTTCAGGC-3′	5′-CTCTCTCAGCGCTGTCATCC-3′	97	XM_005684042.2
*COX4I1*	5′-ATGCTCGACATGAAGGTGGC-3′	5′-GCCTCACTTCTTCCACTCGT-3′	81	XM_018061817.1
*FADD*	5′-AACCACGCGTCACAAGTTTG-3′	5′-AGCCTCTTCAGCACATCACC-3′	82	XM_018043148.1
*ACOX1*	5′-GGCATCGCAGATCCTGATGA-3′	5′-GTGAAGATCCAGAGGCCCAG-3′	78	XM_018063769.1
*PTGS1*	5′-ATCCACTTTCTGCTGACGCA-3′	5′-GGAACGCACTGTGAGTACCA-3′	99	XM_005687044.3
*PTGS2*	5′-CCATGGGTGTGAAAGGGAGG-3′	5′-ATTTGTGCCCTGGGGATCAG-3′	101	XM_018060731.1
*AKT2*	5′-CGGACCCCATGGACTACAAG-3′	5′-CTAACCGCCACCTCCATCTC-3′	73	XM_018062407.1
*AGPAT1*	5′-TTGCCTCTCCCTCATCCTCA-3′	5′-GGGAGAGAAGACCACAACGG-3′	97	NM_001285761.1
*AGPAT2*	5′-TAAGATCGGCCTGTACTGCG-3′	5′-TGCTCATGTTCTCCACCGTC-3′	101	XM_018056130.1
*AGPAT3*	5′-TGGCTGGACGTGGTACTTTC-3′	5′-ATTCGGGGTAGTTGGCCAAG-3′	107	XM_018051810.1
*AGPAT4*	5′-TATCTGCGGTTCGTGCTGTT-3′	5′-AACGAGGGAGAGACAGAGGG-3′	80	XM_005684958.3
*AGPAT5*	5′-CAAGTGGCTCCCGCTGTATG-3′	5′-GTAGCGCTGCAGCTTTTTCC-3′	109	XM_018042052.1

Glyceraldehyde 3-Phosphate Dehydrogenase (*GAPDH*), Actin Beta (*ACTB*), Hypo-xanthine Phosphoribosyl Transferase (*HPRT*), Stearoyl-CoA Desaturase (*SCD*), Acetyl-CoA Carboxylase Alpha (*ACACA*), Fatty Acid Synthase (*FASN*), Lipoprotein Lipase (*LPL*), Leptin (*LEP*), elongation of Very-long-chain Fatty Acid-like Fatty Acid Elongase 1, 2, 3, 4, 5, 6, and 7 (*ELOVL1*, *ELOVL2*, *ELOVL3*, *ELOVL4*, *ELOVL5*, *ELOVL6*, and *ELOVL7*), Carbonyl reductase [NADPH] 2 (*CBR2*), Epoxide Hydrolase 2 (*EPHX2*), Cytochrome C Oxidase Subunit 4I1 (*COX4I1*), Fas Associated via Death Domain (*FADD*), Acyl-CoA Oxidase 1 (*ACOX1*), Prostaglandin-Endoperoxide Synthase 1 and 2 (*PTGS1* and *PTGS2*), AKT Serine/Threonine Kinase 2 (*AKT2*), and 1-Acylglycerol-3-Phosphate O-Acyltransferase 1, 2, 3, 4, and 5 (*AGPAT1*, *AGPAT2*, *AGPAT3*, *AGPAT4*, and *AGPAT5*).

**Table 3:** Feed intake on a fresh matter basis (Kg/goat) and nutrient consumption (g) of the four groups (20 HF, 20 HG, 40 HF, and 40 HG) of goats involved in the trials.

	Treatment			
	20 HF	20 HG	40 HF	40 HG
Diet consumption in kg/goat/day (% of the offered quantity)				
Alfalfa Hay	1.2 (100)	0.7 (100)	1.2 (100)	0.7 (100)
Wheat Straw	0.2 (66)	0.18 (99)	0.15 (50)	0.16 (90)
Concentrate	0.97 (97)	1.29 (99)	0.84 (84)	1.09 (84)
*Schizochytrium* spp. (g)	19.3 (97)	19.8 (99)	33.7 (84)	33.2 (83)
Forage-to-Concentrate Ratio	1.4:0.97 (59:41)	0.88:1.29 (40:60)	1.35:0.84 (61:39)	0.76:1.09 (41:59)
Nutrient intake in g/goat/day (except as noted)				
Dry Matter	2161	1980	2010	1788
Ash	179	144	173	131
Crude Protein	305	309	286	276
Protein Digestible in the Small Intestine (PDI)	190	184	176	162
Ether Extract	79	87	76	83
Ash-free NDF Amylase Treated	853	709	788	649
Acid Detergent Fiber	555	398	515	383
Non Fibrous Carbohydrate	954	920	866	810
Starch	460	538	393	459
NDF/Starch Ratio	1.9	1.3	2.0	1.4
NE Lactation (MJ/day)	11.9	12.2	11	10.9

Numbers in parenthesis represent the percentage (%) of consumed quantities compared to given; 20 HF: goats fed with 20 g *Schizochytrium* spp./day followed a high-forage (F:C = 60:40) diet; 20 HG: goats fed with 20 g *Schizochytrium* spp./day followed a high-grain (F:C = 40:60) diet; 40 HF: goats fed with 40 g *Schizochytrium* spp./day followed a high-forage (F:C = 60:40) diet; 40 HG: goats fed with 40 g *Schizochytrium* spp./day followed a high-grain (F:C = 40:60) diet. PDI has been calculated according to INRA 2018.

**Table 4:** Effect of forage-to-concentrate ratio (F:C) and microalgae inclusion level (ML), and their interaction, on the relative expression of key genes implicated in lipid metabolism of adipose tissue in goats’ tail fat.

	F:C			ML			Effects		
	60:40	40:60	SEM	20 g	40 g	SEM	F:C	ML	F:C × ML
*ACACA*	1.061	0.861	0.062	1.130	0.792	0.062	0.041	0.002	0.005
*ACOX1*	0.969	1.143	0.078	1.019	1.092	0.078	0.140	0.520	0.289
*AGPAT1*	0.936	1.012	0.080	1.000	0.947	0.080	0.516	0.649	0.210
*AGPAT2*	1.352	0.628	0.183	1.079	0.901	0.183	0.016	0.504	0.168
*AGPAT3*	1.293	0.596	0.133	1.094	0.795	0.133	0.003	0.140	0.240
*AGPAT4*	1.071	0.657	0.190	1.059	0.669	0.190	0.150	0.173	0.260
*AGPAT5*	1.067	0.891	0.080	1.024	0.934	0.080	0.144	0.439	0.037
*AKT2*	1.134	0.761	0.141	1.060	0.835	0.141	0.086	0.281	0.407
*CBR2*	0.979	0.875	0.091	1.099	0.755	0.091	0.435	0.021	0.618
*COX4I1*	1.100	0.951	0.054	1.185	0.867	0.054	0.075	0.001	0.622
*ELOVL1*	1.001	1.139	0.092	1.010	1.130	0.092	0.312	0.378	0.217
*ELOVL2*	1.296	0.846	0.255	1.326	0.817	0.255	0.236	0.183	0.376
*ELOVL3*	1.390	0.590	0.295	1.400	0.580	0.295	0.079	0.073	0.602
*ELOVL4*	0.866	1.342	0.179	0.992	1.216	0.179	0.085	0.396	0.299
*ELOVL5*	1.254	0.770	0.126	1.314	0.710	0.126	0.019	0.005	0.424
*ELOVL6*	1.450	0.570	0.161	1.240	0.779	0.161	0.002	0.065	0.352
*ELOVL7*	1.220	0.860	0.279	1.497	0.583	0.279	0.381	0.039	0.601
*EPHX2*	1.157	0.714	0.100	1.002	0.869	0.100	0.009	0.366	0.225
*FADD*	1.053	1.068	0.210	1.020	1.101	0.210	0.960	0.788	0.445
*FASN*	1.188	0.516	0.130	0.967	0.737	0.130	0.003	0.235	0.140
*LEP*	1.145	0.897	0.139	1.250	0.792	0.139	0.231	0.038	0.120
*LPL*	1.107	0.838	0.163	0.909	1.037	0.163	0.265	0.589	0.185
*PTGS1*	0.824	0.956	0.126	0.940	0.840	0.126	0.474	0.583	0.837
*PTGS2*	1.179	1.095	0.160	1.165	1.109	0.160	0.719	0.810	0.060
*SCD*	1.246	0.772	0.074	1.190	0.828	0.074	0.001	0.005	0.116

Acetyl-CoA Carboxylase Alpha (*ACACA*), Acyl-CoA Oxidase 1 (*ACOX1*), 1-Acylglycerol-3-Phosphate O-Acyltransferase 1, 2, 3, 4, 5 (*AGPAT1*, *AGPAT2*, *AGPAT3*, *AGPAT4*, *AGPAT5*), AKT Serine/Threonine Kinase 2 (*AKT2*), Carbonyl reductase [NADPH] 2 (*CBR2*), Cytochrome C Oxidase Subunit 4I1 (*COX4I1*), elongation of Very-long-chain Fatty Acid-like Fatty Acid Elongase 1, 2, 3, 4, 5, 6, 7 (*ELOVL1*, *ELOVL2*, *ELOVL3*, *ELOVL4*, *ELOVL5*, *ELOVL6*, *ELOVL7*), Epoxide Hydrolase 2 (*EPHX2*), Fas Associated via Death Domain (*FADD*), Fatty Acid Synthase (*FASN*), Leptin (*LEP*), Lipoprotein Lipase (*LPL*), Prostaglandin-Endoperoxide Synthase 1 and 2 (*PTGS1* and *PTGS2*), and Stearoyl-CoA Desaturase (*SCD*) relative to the geometrical mean of the references genes: Glyceraldehyde 3-Phosphate Dehydrogenase (*GAPDH*), Actin Beta (*ACTB*), and Hypoxanthine Phosphoribosyl Transferase (*HPRT*).

## Data Availability

Data are contained within the article.

## Supplementary Materials

The following supporting information can be downloaded at: https://www.mdpi.com/article/10.3390/ani14223291/s1, Table S1: effect of the four diets (20 HF, 20 HG, 40 HF, and 40 HG) on milk yield and chemical composition; Table S2: effect of the four diets (20 HF, 20 HG, 40 HF, and 40 HG) on the transcript levels of the genes using one-way ANOVA followed by a post hoc analysis; Table S3: Spearman’s correlation (rho values) between the mRNA expression of genes involved in lipid metabolism in goats’ tail fat; Table S4: Spearman’s correlation (rho values) between the mRNA expression of genes involved in lipid metabolism in goats’ tail fat and milk performance (milk yield, fat, protein, and lactose) or its fatty acid profile at first experimental phase; Table S5: Spearman’s correlation (rho values) between the mRNA expression of genes involved in lipid metabolism in goats’ tail fat and milk performance (milk yield, fat, protein, and lactose) or its fatty acid profile at second experimental phase.

## References

[1] Vernon R.G. (1980). Lipid Metabolism in the Adipose Tissue of Ruminant Animals. Prog. Lipid Res..

[2] Bauman D., Griinari J. (2001). Regulation and nutritional manipulation of milk fat: Low-fat milk syndrome. Livest. Prod. Sci..

[3] Sumner J.M., McNamara J.P. (2007). Expression of Lipolytic Genes in the Adipose Tissue of Pregnant and Lactating Holstein Dairy Cattle. J. Dairy Sci..

[4] Bernard L., Leroux C., Chilliard Y. (2008). Expression and Nutritional Regulation of Lipogenic Genes in the Ruminant Lactating Mammary Gland. Adv. Exp. Med. Biol..

[5] Todorčević M., Hodson L. (2015). The Effect of Marine Derived N-3 Fatty Acids on Adipose Tissue Metabolism and Function. JCM.

[6] Clarke S.D. (2001). Polyunsaturated Fatty Acid Regulation of Gene Transcription: A Molecular Mechanism to Improve the Metabolic Syndrome. J. Nutr..

[7] Krupa K.N., Fritz K., Parmar M. (2024). Omega-3 Fatty Acids. StatPearls [Internet].

[8] Bergen W.G., Mersmann H.J. (2005). Comparative aspects of lipid metabolism: Impact on contemporary research and use of animal models. J. Nutr..

[9] Postic C., Dentin R., Denechaud P.D., Girard J. (2007). ChREBP, a transcriptional regulator of glucose and lipid metabolism. Annu. Rev. Nutr..

[10] Schmitz G., Ecker J. (2008). The opposing effects of n-3 and n-6 fatty acids. Prog. Lipid Res..

[11] Li J., Zhang H., Dong Y., Wang X., Wang G. (2021). Omega-3FAs Can Inhibit the Inflammation and Insulin Resistance of Adipose Tissue Caused by HHcy Induced Lipids Profile Changing in Mice. Front. Physiol..

[12] Peet D.J., Turley S.D., Ma W., Janowski B.A., Lobaccaro J.M., Hammer R.E., Mangelsdorf D.J. (1998). Cholesterol and bile acid metabolism are impaired in mice lacking the nuclear oxysterol receptor LXR alpha. Cell.

[13] Corominas J., Ramayo-Caldas Y., Puig-Oliveras A., Estellé J., Castelló A., Alves E., Pena R.N., Ballester M., Folch J.M. (2013). Analysis of porcine adipose tissue transcriptome reveals differences in de novo fatty acid synthesis in pigs with divergent muscle fatty acid composition. BMC Genom..

[14] Bernard L., Leroux C., Bonnet M., Rouel J., Martin P., Chilliard Y. (2005). Expression and nutritional regulation of lipogenic genes in mammary gland and adipose tissues of lactating goats. J. Dairy Res..

[15] Joseph S.J., Robbins K.R., Pavan E., Pratt S.L., Duckett S.K., Rekaya R. (2010). Effect of Diet Supplementation on the Expression of Bovine Genes Associated with Fatty Acid Synthesis and Metabolism. Bioinform. Biol. Insights.

[16] Vargas-Bello-Pérez E., Bionaz M., Sciarresi-Arechabala P., Cancino-Padilla N., Morales M.S., Romero J., Leskinen H., Garnsworthy P.C., Loor J.J. (2019). Long-Term Effects of Dietary Olive Oil and Hydrogenated Vegetable Oil on Expression of Lipogenic Genes in Subcutaneous Adipose Tissue of Dairy Cows. Vet. Sci..

[17] Mavrommatis A., Tsiplakou E., Zerva A., Pantiora P.D., Georgakis N.D., Tsintzou G.P., Madesis P., Labrou N.E. (2023). Microalgae as a Sustainable Source of Antioxidants in Animal Nutrition, Health and Livestock Development. Antioxidants.

[18] Puri M., Gupta A., Sahni S. (2023). *Schizochytrium* sp.. Trends Microbiol..

[19] Zisis F., Kyriakaki P., Satolias F.F., Mavrommatis A., Simitzis P.E., Pappas A.C., Surai P.F., Tsiplakou E. (2022). The Effect of Dietary Inclusion of Microalgae *Schizochytrium* spp. on Ewes’ Milk Quality and Oxidative Status. Foods.

[20] Mavrommatis A., Tsiplakou E. (2020). The impact of the dietary supplementation level with *Schizochytrium* sp., on milk chemical composition and fatty acid profile of both blood plasma and milk of goats. Small Rumin. Res..

[21] Mavrommatis A., Sotirakoglou K., Kamilaris C., Tsiplakou E. (2021). Effects of Inclusion of *Schizochytrium* spp. and Forage-toConcentrate Ratios on Goats’ Milk Quality and Oxidative Status. Foods.

[22] Franklin S.T., Martin K.R., Baer R.J., Schingoethe D.J., Hippen A.R. (1999). Dietary marine algae (*Schizochytrium* sp.) increases concentrations of conjugated linoleic, docosahexaenoic and transvaccenic acids in milk of dairy cows. J. Nutr..

[23] Meale S.J., Chaves A.V., He M.L., McAllister T.A. (2014). Dose-response of supplementing marine algae (*Schizochytrium* spp.) on production performance, fatty acid profiles, and wool parameters of growing lambs. J. Anim. Sci..

[24] Shi H., Zhang J., Li S., Ji S., Cao Z., Zhang H., Wang Y. (2018). Effects of a Wide Range of Dietary Forage-to-Concentrate Ratios on Nutrient Utilization and Hepatic Transcriptional Profiles in Limit-Fed Holstein Heifers. BMC Genom..

[25] Gao Z., Raza S.H.A., Ma B., Wang Z., Mubarak Alwutayd K., Al Abdulmonem W., Mesfer Alharbi Y., Aljohani A.S.M., Hou S., Gui L. (2023). Effects of Dietary Forage-to-Concentrate Ratio on Fat Deposition, Fatty Acid Composition, Oxidative Stability and mRNA Expression of Sirtuins Genes of Subcutaneous Fat in Sheep (*Ovis Aries*). J. Appl. Anim. Res..

[26] Mavrommatis A., Skliros D., Sotirakoglou K., Flemetakis E., Tsiplakou E. (2021). The Effect of Forage-to-Concentrate Ratio on *Schizochytrium* Spp.-Supplemented Goats: Modifying Rumen Microbiota. Animals.

[27] National Research Council (2007). Nutrient Requirements of Small Ruminants: Sheep, Goats, Cervids. and New World Camelids.

[28] Kyriakaki P., Zisis F., Pappas A.C., Mavrommatis A., Tsiplakou E. (2022). Effects of PUFA-Rich Dietary Strategies on Ruminants’ Mammary Gland Gene Network: A Nutrigenomics Review. Metabolites.

[29] Pfaffl M.W., Tichopad A., Prgomet C., Neuvians T.P. (2004). Determination of Stable Housekeeping Genes, Differentially Regulated Target Genes and Sample Integrity: BestKeeper—Excel-Based Tool Using Pair-Wise Correlations. Biotechnol. Lett..

[30] Allen M.S. (2000). Effects of diet on short-term regulation of feed intake by lactating dairy cattle. J. Dairy Sci..

[31] Baumont R. (1996). Palatability and feeding behaviour in ruminants. A Review. Ann. Zootech..

[32] Jones B.H., Maher M.A., Banz W.J., Zemel M.B., Whelan J., Smith P.J., Moustaid N. (1996). Adipose Tissue Stearoyl-CoA Desaturase mRNA Is Increased by Obesity and Decreased by Polyunsaturated Fatty Acids. Am. J. Physiol.-Endocrinol. Metab..

[33] Rangan V.S., Smith S. (2002). Chapter 6 Fatty Acid Synthesis in Eukaryotes. New Comprehensive Biochemistry.

[34] Jump D.B. (2002). Dietary Polyunsaturated Fatty Acids and Regulation of Gene Transcription. Curr. Opin. Lipidol..

[35] Daniel Z.C.T.R., Wynn R.J., Salter A.M., Buttery P.J. (2004). Differing Effects of Forage and Concentrate Diets on the Oleic Acid and Conjugated Linoleic Acid Content of Sheep Tissues: The Role of Stearoyl-CoA Desaturase1,2. J. Anim. Sci..

[36] Vahmani P., Glover K.E., Fredeen A.H. (2014). Effects of Pasture versus Confinement and Marine Oil Supplementation on the Expression of Genes Involved in Lipid Metabolism in Mammary, Liver, and Adipose Tissues of Lactating Dairy Cows. J. Dairy Sci..

[37] Thering B.J., Graugnard D.E., Piantoni P., Loor J.J. (2009). Adipose Tissue Lipogenic Gene Networks Due to Lipid Feeding and Milk Fat Depression in Lactating Cows. J. Dairy Sci..

[38] Waters S.M., Kelly J.P., O’Boyle P., Moloney A.P., Kenny D.A. (2009). Effect of Level and Duration of Dietary N-3 Polyunsaturated Fatty Acid Supplementation on the Transcriptional Regulation of Δ9-Desaturase in Muscle of Beef Cattle1. J. Anim. Sci..

[39] Urrutia O., Mendizabal J.A., Insausti K., Soret B., Purroy A., Arana A. (2016). Effects of Addition of Linseed and Marine Algae to the Diet on Adipose Tissue Development, Fatty Acid Profile, Lipogenic Gene Expression, and Meat Quality in Lambs. PLoS ONE.

[40] Fan Y., Ren C., Meng F., Deng K., Zhang G., Wang F. (2019). Effects of Algae Supplementation in High-Energy Dietary on Fatty Acid Composition and the Expression of Genes Involved in Lipid Metabolism in Hu Sheep Managed under Intensive Finishing System. Meat Sci..

[41] Alvarenga T.I.R.C., Chen Y., Lewandowski P., Ponnampalam E.N., Sadiq S., Clayton E.H., Van De Ven R.J., Perez J.R.O., Hopkins D.L. (2016). The Expression of Genes Encoding Enzymes Regulating Fat Metabolism Is Affected by Maternal Nutrition When Lambs Are Fed Algae High in Omega-3. Livest. Sci..

[42] Coleman D.N., Carranza Martin A.C., Jin Y., Lee K., Relling A.E. (2019). Prepartum Fatty Acid Supplementation in Sheep. IV. Effect of Calcium Salts with Eicosapentaenoic Acid and Docosahexaenoic Acid in the Maternal and Finishing Diet on Lamb Liver and Adipose Tissue during the Lamb Finishing Period1. J. Anim. Sci..

[43] Coleman D.N., Murphy K.D., Relling A.E. (2018). Prepartum Fatty Acid Supplementation in Sheep. II. Supplementation of Eicosapentaenoic Acid and Docosahexaenoic Acid during Late Gestation Alters the Fatty Acid Profile of Plasma, Colostrum, Milk and Adipose Tissue, and Increases Lipogenic Gene Expression of Adipose Tissue1. J. Anim. Sci..

[44] Chilliard Y., Delavaud C., Bonnet M. (2005). Leptin Expression in Ruminants: Nutritional and Physiological Regulations in Relation with Energy Metabolism. Domest. Anim. Endocrinol..

[45] Coleman D.N., Rivera-Acevedo K.C., Relling A.E. (2018). Prepartum Fatty Acid Supplementation in Sheep I. Eicosapentaenoic and Docosahexaenoic Acid Supplementation Do Not Modify Ewe and Lamb Metabolic Status and Performance through Weaning. J. Anim. Sci..

[46] Al-Shuhaib M.B.S., Al-Thuwaini T.M. (2019). The Effects of Grass-Based versus Grain-Based Feeding of Ruminants on the Human Hygienic Status, a Review. jwpr.

[47] Kyriakaki P., Mavrommatis A., Mitsiopoulou C., Tsiplakou E. (2024). Effect of whole sesame seeds dietary inclusion levels on transcriptional signatures of lipid metabolism in mammary gland of goats. Small Rumin. Res..

