# Supplementary material for: The Interaction of Microalgae Dietary Inclusion and Forage-to-Concentrate Ratio on the Lipid Metabolism-Related Gene Expression in Subcutaneous Adipose Tissue of Dairy Goats
Source: Animals (Basel). 2024 Nov 15;14(22):3291. doi: 10.3390/ani14223291 (PMC11591094; doi:10.3390/ani14223291)
Supplement: Supplementary file 1 [file animals-14-03291-s001.zip › animals-3248066-supplementary.pdf]

Table S1. Effect of the four diets (20HF, 20HG, 40HF and 40HG) on milk yield and chemical composition.

	DIET					TIME							EFFECT		
	20HF	20HG	40HF	HG	SEM	1	2	3	4	5	6	SEM	D	T	DxT
Milk yield (g/d)	2033^{a}	1719^{ab}	1746^{ab}	1386^{b}	224.39	1696^{ac}	1790^{b}	1735^{ab}	1781^{b}	1700^{ad}	1623^{c}	114.83	*	***	*
Fcm$_{4\%}$ (g/ day)	1770^{a}	1611^{ab}	1446^{ab}	1222^{b}	165.88	1534^{a}	1537^{a}	1548^{a}	1548^{a}	1494^{a}	1412^{b}	85.83	*	*	*
ECM (g/day)	1150^{a}	1041^{ab}	950^{ab}	798^{b}	107.39	994^{a}	1006^{a}	1003^{a}	1009^{a}	973^{a}	921^{b}	55.94	*	*	*
Fat (%)	3.37	3.65	3.17	3.30	0.23	3.44^{a}	3.18^{b}	3.46^{a}	3.38^{a}	3.42^{a}	3.31^{ab}	0.13	NS	**	*
Fat yield (g/d)	63.81^{a}	61.57^{a}	49.84^{ab}	44.50^{b}	5.47	57.09^{a}	54.76^{b}	56.93^{ab}	55.67^{ab}	54.24^{ab}	50.88^{c}	2.95	*	**	***
Protein (%)	2.89	3.09	2.94	3.03	0.13	3.00^{a}	2.95^{b}	2.97^{ab}	2.96^{ab}	3.04^{ab}	3.02^{ab}	0.68	NS	*	NS
Protein yield (g/d)	56.83^{a}	51.51^{ab}	48.29^{ab}	40.34^{b}	5.35	49.19^{a}	50.93^{b}	49.63^{ab}	50.65^{ab}	48.83^{ac}	46.21^{d}	2.77	*	***	***
Lactose (%)	4.81^{a}	4.93^{a}	4.53^{b}	4.59^{b}	0.08	4.79^{a}	4.79^{ac}	4.82^{a}	4.67^{b}	4.71^{bc}	4.53^{d}	0.04	*	***	***

20HF: goats fed with 20g *Schizochytrium* spp./day followed a high forage (F:C=60:40) diet; 20HG: goats fed with 20g *Schizochytrium* spp./day followed a high grain (F:C=40:60) diet; 40HF: goats fed with 40g *Schizochytrium* spp./day followed a high forage (F:C=60:40) diet; 40HG: goats fed with 40g *Schizochytrium* spp./day followed a high grain (F:C=40:60) diet; FCM4% = (0.40 + 0.15 × F) × M, where F = fat content (%) and M = milk yield in kg; Energy corrected milk (ECM) yield based on the Equation, ECM = (milk yield × 0.327) + (fat yield × 12.95) + (protein yield × 7.2).

Table S2. Effect of the four diets (20HF, 20HG, 40HF and 40HG) on the transcript levels of the genes using one-way ANOVA followed by a post hoc analysis.

	**20HF**	**20HG**	**40HF**	**40HG**	**SEM**	**P-value**
ACACA	1.215^{a}	1.081^{a}	0.983^{ab}	0.735^{b}	0.063	0.057
ACOX1	0.872^{a}	0.905^{a}	1.062^{ab}	1.167^{b}	0.047	0.098
AGPAT1	0.914	1.005	1.128	0.936	0.040	0.240
AGPAT2	1.327^{a}	1.238^{a}	0.928^{ab}	0.564^{b}	0.099	0.018
AGPAT3	1.328^{a}	1.158^{ab}	0.715^{b}	0.806^{b}	0.087	0.043
AGPAT4	1.226^{a}	1.033^{a}	1.232^{a}	0.528^{b}	0.095	0.024
AGPAT5	1.129	0.927	0.947	1.023	0.038	0.261
AKT2	1.142	1.036	1.103	0.762	0.070	0.205
CBR2	1.182^{a}	0.765^{b}	1.160^{a}	0.928^{ab}	0.061	0.038
COX4I1	1.127	0.944	1.040	0.930	0.033	0.130
ELOVL1	0.916	0.966	1.051	1.050	0.048	0.726
ELOVL2	1.402	0.757	1.132	0.790	0.131	0.288
ELOVL3	1.746^{a}	1.131^{a}	0.999^{a}	0.206^{b}	0.157	0.005
ELOVL4	0.888	0.791	1.234	1.072	0.086	0.251
ELOVL5	1.336^{a}	1.032^{a}	1.255^{a}	0.423^{b}	0.110	0.009
ELOVL6	1.372^{a}	1.262^{ab}	0.858^{bc}	0.535^{c}	0.096	0.005
ELOVL7	1.323	0.916	1.235	0.598	0.122	0.122
EPHX2	1.297^{a}	1.133^{a}	1.018^{a}	0.655^{b}	0.072	0.006
FADD	1.194	0.809	1.003	1.033	0.081	0.438
FASN	1.309^{a}	1.221^{a}	0.940^{ab}	0.510^{b}	0.100	0.017
LEP	1.369^{a}	1.064^{a}	0.991^{ab}	0.610^{b}	0.089	0.025

LPL	1.093	0.826	1.106	1.014	0.089	0.672
PTGS1	1.002[ab]	0.857[b]	1.257[a]	0.885[b]	0.061	0.065
PTGS2	1.125	0.851	0.853	1.181	0.069	0.179
SCD	1.324[a]	1.209[ab]	0.877[bc]	0.625[c]	0.079	0.003

20HF: goats fed with 20g *Schizochytrium* spp./day followed a high forage (F:C=60:40) diet; 20HG: goats fed with 20g *Schizochytrium* spp./day followed a high grain (F:C=40:60) diet; 40HF: goats fed with 40g *Schizochytrium* spp./day followed a high forage (F:C=60:40) diet; 40HG: goats fed with 40g *Schizochytrium* spp./day followed a high grain (F:C=40:60) diet.

Table S3. Spearman correlation (rho values) between the mRNA expression of genes involved in lipid metabolism in goats' tail fat.

	ACACA	*ACOX1*	*AGPAT1*	*AGPAT2*	*AGPAT3*	*AGPAT4*	*AGPAT5*	*AKT2*	*CBR2*	*COX4I1*	*ELOVL1*	*ELOVL2*	*ELOVL3*	*ELOVL4*	*ELOVL5*	*ELOVL6*	*ELOVL7*	*EPHX2*	*FADD*	*FASN*	*LEP*	*LPL*	*PTGS1*	*PTGS2*
ACOX1	-0.090																							
AGPAT1	0.014	0.111																						
AGPAT2	0.742	-0.144	-0.048																					
AGPAT3	0.637	-0.221	0.010	0.834																				
AGPAT4	0.765	-0.008	0.254	0.671	0.624																			
AGPAT5	0.014	0.105	0.163	0.057	0.254	0.279																		
AKT2	0.725	0.103	0.136	0.770	0.723	0.701	0.093																	
CBR2	0.268	0.287	0.126	0.202	0.170	0.401	0.276	0.393																
COX4I1	0.449	-0.041	0.140	0.429	0.504	0.351	0.279	0.470	0.065															
ELOVL1	-0.239	0.668	0.083	-0.211	-0.399	0.011	0.029	-0.108	0.319	-0.285														
ELOVL2	-0.291	0.108	0.156	-0.467	-0.347	-0.066	0.071	-0.287	0.113	-0.078	0.110													
ELOVL3	0.755	-0.201	-0.145	0.715	0.774	0.668	0.033	0.554	0.098	0.518	-0.397	-0.194												
ELOVL4	-0.136	0.119	0.034	-0.458	-0.514	-0.237	-0.181	-0.214	-0.095	-0.334	-0.022	0.276	-0.331											
ELOVL5	0.875	0.006	0.122	0.779	0.687	0.734	-0.030	0.733	0.214	0.648	-0.171	-0.070	0.775	-0.240										
ELOVL6	0.700	-0.344	-0.248	0.752	0.698	0.632	0.174	0.566	0.015	0.551	-0.411	-0.126	0.778	-0.287	0.809									
ELOVL7	0.628	-0.163	-0.043	0.587	0.718	0.537	0.183	0.568	0.135	0.737	-0.429	-0.289	0.781	-0.235	0.677	0.661								
EPHX2	0.782	-0.258	-0.105	0.793	0.738	0.557	0.025	0.778	0.103	0.584	-0.273	-0.262	0.683	-0.289	0.844	0.839	0.638							
FADD	0.091	0.606	0.070	0.130	-0.083	0.303	0.228	0.313	0.624	-0.225	0.587	0.163	-0.179	-0.054	0.099	-0.129	-0.296	-0.014						
FASN	0.796	-0.028	-0.056	0.877	0.745	0.750	0.125	0.829	0.419	0.408	-0.079	-0.308	0.644	-0.278	0.805	0.683	0.560	0.867	0.293					
LEP	0.793	-0.038	0.079	0.729	0.675	0.675	0.074	0.617	0.179	0.300	-0.187	-0.305	0.712	-0.191	0.700	0.515	0.617	0.536	0.054	0.598				
LPL	0.097	0.403	0.024	0.191	0.040	0.303	0.512	0.298	0.498	-0.020	0.277	0.097	-0.145	0.071	0.082	0.090	-0.106	0.084	0.635	0.419	0.046			
PTGS1	-0.013	0.084	0.352	-0.248	-0.411	0.129	0.047	-0.177	0.361	-0.226	0.390	0.192	-0.306	0.363	-0.122	-0.348	-0.254	-0.269	0.228	-0.069	-0.104	0.258		
PTGS2	0.115	0.398	-0.081	-0.096	-0.141	0.187	0.236	0.131	0.310	-0.110	0.319	0.265	-0.194	0.175	-0.008	-0.067	-0.182	-0.172	0.595	0.122	0.107	0.568	0.164	
SCD	0.836	-0.154	-0.181	0.741	0.595	0.673	0.093	0.590	0.173	0.404	-0.216	-0.237	0.752	-0.145	0.800	0.779	0.636	0.758	-0.010	0.779	0.646	0.153	0.031	0.057

Table S4. Spearman correlation (rho values) between the mRNA expression of genes involved in lipid metabolism in goats' tail fat and milk performance (milk yield, fat, protein, and lactose) or its fatty acid profile at first experimental phase.

	ACACA	*ACOX1*	*AGPAT1*	*AGPAT2*	*AGPAT3*	*AGPAT4*	*AGPAT5*	*AKT2*	*CBR2*	*COX4I1*	*ELOVL1*	*ELOVL2*	*ELOVL3*	*ELOVL4*	*ELOVL5*	*ELOVL6*	*ELOVL7*	*EPHX2*	*FADD*	*FASN*	*LEP*	*LPL*	*PTGS1*	*PTGS2*	*SCD*
**Milk yield (g)**	0.170	-0.148	0.212	0.482	0.554	0.587	0.234	0.319	0.079	0.256	0.006	0.077	0.358	-0.624	0.494	0.354	0.109	0.182	0.319	0.279	0.176	0.211	-0.395	0.430	-0,081
**FCM 4% (g)**	0.267	-0.100	0.290	0.529	0.536	0.630	0.296	0.267	0.015	0.224	-0.019	0.083	0.414	-0.676	0.500	0.354	0.124	0.165	0.349	0.248	0.311	0.228	-0.329	0.375	0,002
**ECM (g)**	0.253	-0.143	0.273	0.511	0.571	0.625	0.300	0.289	0.015	0.206	-0.041	0.051	0.451	-0.674	0.474	0.335	0.144	0.103	0.313	0.223	0.325	0.206	-0.319	0.362	0,005
**Fat (%)**	0.051	0.098	0.053	0.009	-0.412	-0.264	-0.271	-0.137	-0.255	-0.571	-0.067	-0.322	-0.328	0.360	-0.524	-0.263	-0.285	-0.209	-0.149	-0.066	0.201	-0.017	0.261	-0.348	0,221
**Fat (g)**	0.197	-0.115	0.228	0.511	0.434	0.591	0.251	0.216	-0.051	0.091	-0.055	0.097	0.333	-0.575	0.374	0.311	0.079	0.015	0.338	0.176	0.350	0.252	-0.249	0.362	-0,007
**Protein (%)**	-0.103	-0.119	-0.071	-0.344	-0.209	-0.348	-0.317	-0.047	-0.117	-0.529	-0.152	-0.506	-0.216	0.472	-0.715	-0.445	-0.041	-0.259	-0.403	-0.245	0.090	-0.360	0.340	-0.337	-0,002
**Protein (g)**	0.182	-0.294	0.228	0.381	0.598	0.591	0.317	0.294	-0.007	0.224	-0.160	0.062	0.493	-0.579	0.350	0.321	0.156	0.029	0.181	0.091	0.280	0.127	-0.271	0.337	-0,029
**Lactose (%)**	-0.021	-0.080	-0.099	0.145	0.018	0.053	-0.008	0.032	-0.460	0.141	-0.096	0.151	0.276	-0.428	0.495	0.362	0.165	0.405	0.096	0.045	-0.176	-0.264	-0.500	-0.068	-0,249
**SCFA**	0.085	0.443	-0.032	-0.205	-0.319	0.006	-0.042	-0.248	0.232	-0.218	0.376	0.087	-0.279	0.212	-0.318	-0.375	-0.226	-0.179	0.086	0.049	0.003	0.015	0.441	0.049	0,272
**MCFA**	0.010	0.051	0.135	-0.092	-0.286	0.396	0.212	0.010	0.209	-0.091	0.159	0.413	-0.265	0.104	-0.012	0.007	-0.482	-0.106	0.415	0.127	-0.224	0.353	0.437	0.162	-0,123
**LCFA**	0.019	-0.187	0.181	-0.059	0.063	-0.151	-0.075	0.363	-0.416	0.374	-0.167	-0.209	0.044	-0.067	0.376	0.170	0.394	0.347	-0.269	-0.042	-0.127	-0.395	-0.395	0.015	-0,051
**MUFA**	0.028	-0.154	-0.330	0.389	0.292	-0.259	-0.033	-0.358	-0.129	-0.387	-0.262	-0.338	0.346	-0.271	-0.138	0.150	0.082	-0.126	-0.267	0.000	0.350	0.112	-0.315	-0.475	0,315
**PUFA**	-0.100	-0.574	-0.514	0.247	0.441	-0.034	-0.045	-0.033	0.097	-0.428	-0.414	-0.029	0.265	0.023	-0.113	0.354	-0.222	-0.085	-0.178	-0.107	0.179	-0.032	-0.179	-0.094	0,045
**SFA**	0.001	0.290	0.395	-0.449	-0.366	0.204	0.015	0.306	0.009	0.424	0.301	0.217	-0.316	0.245	0.076	-0.288	0.094	0.106	0.200	-0.032	-0.311	-0.223	0.340	0.366	-0,238
**UFA**	-0.020	-0.290	-0.389	0.430	0.360	-0.211	-0.030	-0.316	-0.020	-0.479	-0.305	-0.233	0.292	-0.238	-0.124	0.271	-0.118	-0.112	-0.204	0.015	0.302	0.213	-0.323	-0.368	0,203
**SFA/ UFA**	0.013	0.298	0.399	-0.442	-0.371	0.209	0.018	0.310	0.027	0.459	0.313	0.242	-0.305	0.250	0.107	-0.286	0.097	0.100	0.206	-0.026	-0.301	-0.216	0.339	0.371	-0,216
**AI**	-0.104	0.171	0.299	-0.345	-0.312	0.314	0.188	0.234	0.020	0.364	0.248	0.367	-0.294	0.118	0.124	-0.037	-0.047	0.076	0.298	-0.059	-0.373	-0.093	0.259	0.359	-0,360
**TI**	0.104	-0.017	0.321	-0.110	-0.136	-0.088	-0.030	0.389	-0.358	0.369	-0.050	-0.307	-0.142	0.084	0.163	-0.005	0.401	0.312	-0.165	0.031	-0.090	-0.339	-0.088	-0.002	0,115
**HPI**	0.014	-0.329	-0.110	0.101	0.205	-0.338	-0.240	-0.139	-0.035	-0.328	-0.329	-0.040	0.188	0.056	-0.135	0.002	-0.028	-0.191	-0.339	-0.211	0.240	0.014	0.078	-0.411	0,427
**ω6**	-0.181	-0.370	-0.009	0.034	0.321	-0.202	-0.342	0.191	-0.209	-0.097	-0.301	-0.119	0.191	0.003	-0.079	0.127	0.109	0.003	-0.405	-0.316	0.036	-0.439	-0.453	-0.061	-0,159
**ω3**	-0.290	-0.298	-0.089	-0.157	0.386	0.101	0.242	-0.118	0.178	0.224	-0.171	0.468	0.412	-0.200	0.179	0.204	0.065	-0.268	-0.055	-0.370	-0.035	0.028	-0.065	0.119	-0,392
**ω6/ω3**	0.109	0.065	-0.015	0.280	0.061	-0.239	-0.453	0.228	-0.245	-0.332	-0.026	-0.475	0.000	0.013	-0.174	-0.094	0.115	0.147	-0.152	0.069	0.280	-0.412	-0.288	-0.193	0,199
**EPA**	-0.427	-0.045	-0.288	-0.165	-0.237	-0.251	-0.064	-0.423	-0.081	-0.492	0.091	0.291	-0.149	-0.052	-0.272	0.126	-0.574	-0.272	-0.173	-0.210	-0.414	-0.068	0.019	-0.374	-0,030
**DPA**	-0.138	-0.376	-0.133	0.357	0.430	-0.034	-0.158	0.144	0.049	-0.145	-0.365	-0.053	0.060	0.035	-0.105	0.329	-0.055	0.101	-0.066	-0.086	0.051	0.163	-0.484	0.160	-0,278
**DHA**	-0.078	-0.491	0.049	0.115	0.448	0.315	0.236	0.139	0.086	-0.071	-0.377	0.259	0.293	-0.065	0.134	0.345	-0.191	-0.159	-0.022	-0.293	0.253	0.168	-0.050	0.175	-0,263

Table S5. Spearman correlation (rho values) between the mRNA expression of genes involved in lipid metabolism in goats' tail fat and milk performance (milk yield, fat, protein, and lactose) or its fatty acid profile at second experimental phase.

	ACACA	*ACOX1*	*AGPAT1*	*AGPAT2*	*AGPAT3*	*AGPAT4*	*AGPAT5*	*AKT2*	*CBR2*	*COX4I1*	*ELOVL1*	*ELOVL2*	*ELOVL3*	*ELOVL4*	*ELOVL5*	*ELOVL6*	*ELOVL7*	*EPHX2*	*FADD*	*FASN*	*LEP*	*LPL*	*PTGS1*	*PTGS2*	*SCD*
**Milk yield (g)**	0.282	0.493	0.321	0.205	0.198	0.531	0.122	0.424	0.178	0.185	0.064	0.286	0.534	0.346	0.553	0.321	0.269	0.176	0.232	0.283	0.385	0.126	0.122	0.004	0.263
**FCM 4% (g)**	0.156	0.373	0.365	0.098	0.160	0.458	0.203	0.286	0.149	0.181	0.054	0.332	0.474	0.435	0.396	0.179	0.249	0.065	0.193	0.250	0.317	0.104	0.161	0.058	0.172
**ECM (g)**	0.216	0.340	0.398	0.135	0.181	0.508	0.126	0.317	0.159	0.183	0.041	0.329	0.505	0.429	0.456	0.223	0.273	0.097	0.182	0.277	0.377	0.068	0.204	0.032	0.226
**Fat (%)**	-0.205	-0.214	-0.107	-0.232	-0.100	-0.219	0.099	-0.418	-0.189	-0.199	0.039	-0.044	-0.189	0.230	-0.296	-0.289	-0.032	-0.274	-0.138	-0.015	0.005	0.040	0.008	0.318	-0.135
**Fat (g)**	0.058	0.238	0.343	0.040	0.141	0.377	0.244	0.176	0.153	0.160	-0.006	0.376	0.393	0.500	0.295	0.065	0.244	-0.002	0.198	0.267	0.273	0.161	0.185	0.142	0.088
**Protein (%)**	-0.212	-0.645	0.021	-0.214	-0.117	-0.332	-0.124	-0.265	-0.243	-0.100	-0.219	-0.143	-0.404	-0.104	-0.504	-0.277	-0.184	-0.098	-0.422	-0.257	-0.333	-0.277	-0.021	-0.365	-0.278
**Protein (g)**	0.305	0.381	0.398	0.237	0.232	0.563	0.104	0.454	0.181	0.230	0.029	0.263	0.558	0.339	0.567	0.323	0.321	0.207	0.190	0.321	0.426	0.079	0.150	-0.072	0.278
**Lactose (%)**	-0.007	0.522	0.211	0.149	0.058	0.247	0.240	0.089	0.105	-0.066	0.290	0.024	0.116	0.261	0.255	-0.003	0.075	0.015	0.277	0.249	0.276	0.200	0.147	0.161	0.021
**SCFA**	-0.047	0.364	0.011	0.053	-0.181	0.126	0.241	-0.033	0.267	-0.059	0.090	0.029	-0.054	-0.012	0.198	0.086	-0.074	0.004	0.158	0.081	-0.028	0.354	0.287	0.382	0.053
**MCFA**	0.019	-0.172	0.529	0.196	0.141	0.284	0.305	0.445	0.263	0.406	-0.179	-0.009	0.065	0.014	0.077	0.040	0.179	0.404	-0.131	0.150	-0.075	0.270	0.144	-0.367	-0.148
**LCFA**	0.372	0.075	0.214	0.195	0.362	0.439	-0.290	0.356	-0.034	0.102	-0.035	0.111	0.502	0.332	0.472	0.274	0.288	0.121	0.147	0.373	0.488	-0.167	0.106	-0.072	0.366
**MUFA**	-0.258	0.197	-0.626	-0.404	-0.195	-0.547	-0.074	-0.463	-0.289	-0.453	0.188	0.069	-0.249	-0.093	-0.419	-0.249	-0.307	-0.484	0.131	-0.331	-0.257	-0.207	-0.451	0.207	-0.164
**PUFA**	-0.212	-0.067	-0.550	-0.144	-0.139	-0.553	-0.167	-0.365	-0.193	-0.249	0.056	-0.331	-0.370	-0.353	-0.456	-0.188	-0.244	-0.258	0.025	-0.287	-0.437	-0.200	-0.354	0.005	-0.158
**SFA**	0.314	-0.164	0.618	0.432	0.218	0.595	0.094	0.507	0.308	0.476	-0.193	-0.075	0.323	0.102	0.472	0.300	0.344	0.525	-0.124	0.407	0.309	0.244	0.448	-0.188	0.234
**UFA**	-0.305	0.164	-0.626	-0.426	-0.224	-0.588	-0.090	-0.508	-0.302	-0.463	0.209	0.066	-0.319	-0.105	-0.470	-0.302	-0.333	-0.514	0.120	-0.400	-0.284	-0.242	-0.445	0.191	-0.222
**SFA/ UFA**	0.310	-0.164	0.622	0.429	0.221	0.591	0.092	0.508	0.305	0.470	-0.201	-0.071	0.321	0.104	0.471	0.301	0.339	0.520	-0.122	0.403	0.296	0.243	0.447	-0.190	0.228
**AI**	0.250	-0.157	0.586	0.413	0.219	0.523	0.102	0.481	0.225	0.484	-0.213	-0.059	0.275	0.069	0.398	0.264	0.287	0.502	-0.177	0.273	0.201	0.154	0.369	-0.268	0.147
**TI**	0.288	-0.235	0.407	0.145	0.192	0.514	-0.160	0.226	0.026	0.244	-0.062	0.164	0.372	0.409	0.354	0.120	0.348	0.248	-0.137	0.333	0.495	-0.061	0.307	-0.026	0.261
**HPI**	-0.139	-0.137	-0.096	-0.291	0.160	-0.096	0.130	-0.260	-0.178	-0.182	0.077	0.002	-0.081	0.023	-0.219	-0.100	0.126	-0.362	-0.015	0.075	0.100	-0.043	-0.095	0.130	-0.178
**ω6**	-0.018	0.089	-0.381	-0.015	-0.033	-0.363	-0.271	-0.126	-0.102	-0.127	0.092	-0.077	-0.065	-0.226	-0.133	-0.017	-0.173	-0.182	0.261	-0.195	-0.176	-0.197	-0.260	-0.063	0.017
**ω3**	-0.047	0.047	-0.058	0.018	0.191	-0.316	-0.106	-0.074	-0.025	-0.093	-0.047	-0.323	-0.068	-0.352	-0.194	0.019	-0.113	-0.228	0.133	-0.129	-0.263	-0.154	-0.187	-0.068	-0.011
**ω6/ω3**	-0.081	-0.209	0.056	-0.083	-0.409	0.005	-0.160	-0.001	-0.010	0.027	-0.021	0.369	-0.104	0.297	0.059	-0.188	-0.116	0.223	-0.036	-0.078	-0.030	0.013	0.203	-0.303	-0.129
**EPA**	-0.095	0.231	0.111	0.171	0.183	-0.172	-0.019	-0.110	-0.165	-0.060	0.244	-0.518	-0.109	-0.069	-0.109	-0.135	0.013	-0.072	0.016	0.131	0.026	-0.207	-0.078	-0.155	-0.038
**DPA**	-0.362	0.200	-0.466	-0.288	-0.121	-0.605	0.142	-0.305	-0.083	-0.264	0.140	0.042	-0.318	-0.412	-0.394	-0.234	-0.416	-0.416	0.270	-0.509	-0.414	0.099	-0.394	0.276	-0.318
**DHA**	-0.114	0.199	-0.224	-0.051	0.261	-0.402	0.025	-0.179	-0.168	-0.228	-0.007	-0.280	-0.104	-0.267	-0.251	-0.007	-0.146	-0.349	0.115	-0.201	-0.228	-0.132	-0.367	0.179	-0.065
